# *MicroRNA-613* Enhances Nasopharyngeal Carcinoma Cell Radiosensitivity *via* the *DNA Methyltransferase 3B*/*Tissue Inhibitor of Matrix Metalloproteinase-3*/Signal Transducer and Activator of Transcription-1/Forkhead Box O-1 Axis

**DOI:** 10.1155/2022/5699275

**Published:** 2022-08-26

**Authors:** Liqiang Deng, Qing Yin, Shuyun Liu, Debao Luo

**Affiliations:** ^1^Department of Pediatric Otolaryngology, The First People's Hospital of Chenzhou, Hunan Province 423000, China; ^2^Department of Pediatric Gastroenterology, The First People's Hospital of Chenzhou, Hunan Province 423000, China; ^3^Department of Otolaryngology-Head and Neck Surgery, The Affiliated Hospital of Southwest Medical University, Taiping Avenue 25, Luzhou 646000, China; ^4^Department of Otorhinolaryngology, The First People's Hospital of Chenzhou, Hunan Province 423000, China

## Abstract

Nasopharyngeal carcinoma (NPC) is a common malignancy of the nasopharynx, and radioresistant represents the main obstacle in NPC treatment. Malignant transformation of normal cells is driven by genetic and epigenetic changes, which are primarily manifested as changes in miRNA levels and DNA methylation status. microRNA (miR)-613 plays an inhibitory role in several types of cancer. Herein, the current study sought to explore the roles of *miR-613* in NPC cell radiosensitivity. *miR-613* expression patterns in NPC tissues were detected, and its correlation with clinical indexes was analyzed. NP-69 and C666-1 cell lines were selected for cellular experimentation. Radioresistant cell line C666-1R was obtained by fractionated radiation. Cell viability, survival fraction, and apoptosis were detected by CCK-8, colony formation assay, and flow cytometry. The binding relation between *miR-613* and *DNMT3B* was verified by dual-luciferase and RIP assays. *miR-613* was lowly expressed in NPC tissues and cells, with lower expression levels in C666-1R than C666-1, and further correlated with lymph node metastasis, tumor size, and tumor metastasis. *miR-613* overexpression reduced C666-1R cell viability and survival fraction and increased apoptosis, while C666-1 cells with silencing *miR-613* presented the opposite trends. *miR-613* targeted *DNMT3B*. *miR-613* and *DNMT3B* overexpression led to enhanced C666-1R cell viability and survival fraction and decreased apoptosis. *miR-613* reduced *TIMP3* methylation and elevated *TIMP3* protein level by inhibiting *DNMT3B*. *miR-613* enhanced NPC radiosensitivity by inhibiting the *DNMT3B*/*TIMP3*/STAT1/FOXO1 pathway. Collectively, *miR-613* inhibited *DNMT3B*, reduced *TIMP3* methylation, and increased *TIMP3* protein level, thus inhibiting the STAT1/FOXO1 pathway and enhancing the radiosensitivity of NPC cells.

## 1. Introduction

Nasopharyngeal carcinoma (NPC), defined as a head and neck cancer malignancy arising from the epithelium of the nasopharynx, is associated with bleak prognoses and increased lymphocyte infiltration [1, 2]. Interestingly, NPC has a dramatically skewed geographic distribution across the world, with significantly high incidence in East and Southeast Asia [3]. Epstein Barr virus represents the leading factor in the etiology of NPC; in addition, genetic factors and environmental and dietary causes can also precipitate NPC [4, 5]. Currently, radiotherapy is regarded as the gold-standard treatment for NPC [6]. However, the onset of radiation resistance poses a major hindrance in NPC treatment [7]. Accordingly, preventing radioresistance and increasing radiosensitivity are of the utmost importance for improving NPC patient prognosis and increasing 5-year survival rates [8]. In lieu of the same, it is imperative to expand on the potential mechanism of NPC radiosensitivity, provide novel effective strategies for clinical treatment, and prolong the survival time of patients with NPC.

microRNAs (miRNAs) are well-established as a group of small noncoding RNAs functioning in the posttranscriptional regulation of gene expression, also known for their crucial ability to regulate a variety of cellular activities, such as cell differentiation, growth, apoptosis, and development. Moreover, accumulating studies have indicated the association of miRNAs with various diseases, such that currently ongoing miRNA-mediated clinical trials have shown promising results in the treatment of viral infection and cancer [9]. As an important factor of posttranscriptional regulation, miRNAs are also capable of participating in ionizing radiation responses of cancer cells *via* the regulation of the cell cycle and apoptotic pathways [10–12]. What is more, hundreds of miRNAs have been discovered in diverse animals, and many of these are phylogenetically conserved [13]. miRNAs are 18-25 nucleotide long noncoding RNA molecules with the function of posttranscriptional gene regulation, which have attracted extensive attention as potential cancer regulators and biomarkers. Some miRNAs have tumor-suppressive or carcinogenic effects in oral carcinogenesis [14] and can be used as the biomarker for early oral cancer [15] and screening biomarkers for diagnosis and prognosis of colorectal carcinoma, sessile serrated lesions [16], and renal cell carcinoma patients [17]. Garnering our attention, miRNAs are associated with the malignant biological characteristics of cancer cells, including radiosensitivity [18, 19]. Furthermore, miRNA imbalances can contribute to tumor progression and metastasis. For instance, one such miRNA, namely, *miR-613*, confers an anticancer role in bladder cancer by targeting SphK1 [20]. In addition, *miR-613* exhibits a tumor-suppressive role in several types of cancer [21]. One prior study uncovered that *miR-613* plays an anticancer role in hepatocellular carcinoma by targeting tryptophan 5-monooxygenase activation protein [22]. Similarly, *miR-613* was previously shown to inhibit proliferation, invasion, and tumor formation of retinoblastoma cells by targeting E2F transcription factor 5 [23]. Moreover, *miR-613* can attenuate the Warburg effect in gastric cancer *by* targeting g6-phosphofructo-2-kinase/fructose-2,6-biphosphatase 2 [24]. *miR-613* targets sex-determining region Y-box 9 to play an antitumor role in glioma cells [25]. *miR-613* inhibits the proliferation, migration, and invasion of thyroid papillary carcinoma cells by directly targeting transgelin 2 [26]. On the other hand, knockdown of *miR-613* promotes the proliferation and metastasis of pancreatic cancer cells by facilitating Notch3 expression and regulating the Notch3 pathway [27]. Long noncoding RNA cytoskeleton regulator RNA has also been previously indicated to facilitate cell metastasis and invasion by titrating *miR-613* in NPC [28]. Similarly, the family with sequence similarity 225 member B promotes the proliferation and metastasis of NPC *via* the *miR-613*/cyclin D2 axis [29]. Besides, *miR-613* can further inactivate the AKT signal pathway by targeting fibronectin1, thus exerting antiangiogenic effects on NPC cells [30]. Together, these shreds of evidence highlight the potential involvement of *miR-613* in the progression of NPC. However, whether *miR-613* affects the radiosensitivity of NPC remains largely unknown.

The malignant transformation of normal cells is driven by genetic and epigenetic changes, and the dynamic regulation of DNA methylation serves as a key epigenetic mechanism for the occurrence, maintenance, and progression of cancer [31]. Moreover, epigenetic changes are primarily manifested as changes in miRNA levels and DNA methylation status [32]. *DNA methyltransferase 3B* (*DNMT3B*), as a major DNA methyltransferase, is known to function in *de novo* methylation of DNA, which exerts critical roles in various important biological functions, while also being involved in tumorigenesis, progression, and metastasis through DNA methylation [33–36]. What is noteworthy is that silencing *DNMT3B* restores and activates p53 and p21 through DNA demethylation, ensuing cell cycle arrest and apoptosis, thus improving the radiosensitivity of NPC [37]. *Tissue inhibitor of matrix metalloproteinases-3* (*TIMP3*) is a special member of the *TIMP* family, which possesses the ability to regulate tumor growth, metastasis, angiogenesis, and other physiological progress through matrix metalloproteinases [38]. Furthermore, as an independent and promising biomarker, *TIMP3* plays a regulatory role in a plethora of cancers [39–41] and is lowly expressed in NPC tissues [42]. In addition, *TIMP3* methylation has been documented in several malignancies [34, 43, 44]. Strikingly, inhibition of *DNMT3B* reduces *TIMP3* methylation levels and enhances *TIMP3* protein levels, thus modulating the STAT1/FOXO1 pathway in breast cancer [34]. However, no studies have explored whether *miR-613* regulates the *DNMT3B*/*TIMP3*/STAT1/FOXO1 pathway in the radiosensitivity of NPC cells. Accordingly, the current sets out to investigate the potential molecular mechanism of *miR-613* in NPC cell radiosensitivity by targeting *DNMT3B*, affecting *TIMP3* methylation level, and activating the STAT1/FOXO1 pathway.

## 2. Materials and Methods

### 2.1. Ethics Statement

The current study was approved by the ethics committee of the First People's Hospital of Chenzhou and performed in strict accordance with the code of ethics. Signed informed consents were obtained from all participants after a comprehensive understanding prior to specimen collection.

### 2.2. Sample Collection

A total of 121 pairs of adjacent normal tissues and tumor tissues from NPC patients (including 81 males and 40 females; aged 40-68 years with a mean calculated age of 51.3 ± 7.2 years) were collected at the First People's Hospital of Chenzhou from September 2016 to September 2020. The inclusion criteria [45] were as follows: (1) patients with NPC confirmed by biopsy and pathology; (2) patients who did not receive treatment prior to inclusion; (3) patients without distant metastasis; (4) patients receiving intensity-modulated radiotherapy; and (5) patient with complete clinical data. Patients not meeting any one of the above inclusion criteria were excluded and were not included for sample collection. In addition, none of the included patients received radiotherapy or chemotherapy before operation. According to the tumor, node, and metastasis (TNM) staging [46], there were 21 cases at stage I, 20 cases at stage II, and 80 cases at stages III and IV.

### 2.3. Cell Culture and Grouping

NP-69 and C666-1R cells were procured from ATCC (Manassas, VA, USA). C666-1R cells were prepared by *in vitro* fractionated radiation [47, 48]). All cell lines were cultured in RPMI-1640 medium (22400089, Gibco Company, Grand Island, NY, USA) supplemented with 10% fetal bovine serum and plated into 6-well plates (at a density of 1 × 10^5^ cells/well), and subcultured in a humidified incubator with saturated humidity and 5% CO_2_ at 37°C.

Upon achieving 70-80% confluence, the C666-1R or C666-1 cells were plated into 6-well plates (at a density of 1 × 10^6^ cells/well), cultured in a humidified incubator with saturated humidity and 5% CO_2_ at 37°C, and allocated and treated as according as follows: the NP-69 group, the C666-1 group, the C666-1R group, the C666-1R + miR mimi group (C666-1R transfected with 1 *miR-613* mimics), the C666-1R + mimi NC group (C666-1R transfected with mimics NC), the C666-1 + miR inhi group (C666-1R transfected with *miR-613* inhibitor), the C666-1 + inhi NC group (C666-1R transfected with inhibitor NC), the C666-1R + miR mimi + oe-*DNMT3B* group (C666-1R co-transfected with *miR-613* mimics and oe-*DNMT3B* plasmid), the C666-1R + miR mimi + oe-NC group (C666-1R co-transfected with *miR-613* mimics and oe-NC plasmid), the C666-1R + miR mimi+oe-*DNMT3B* + si-STAT1 group (C666-1R co-transfected with *miR-613* mimics, oe-*DNMT3B* and si-STAT1 plasmid), and the C666-1R + miR mimi + oe-*DNMT3B* + si-NC group (C666-1R co-transfected with *miR-613* mimics, oe-*DNMT3B*, and si-NC plasmid). Further experimentation was carried out after 12 h mimics NC, *miR-613* mimics, oe-NC, oe-*DNMT3B*, si-NC, and si-STAT1 were transfected into cells using the Lipofectamine 2000 reagent (Invitrogen, Burlington, ON, Canada). The final transfection concentration was 100 nM. All the above-utilized plasmids were purchased from the Life Technologies Corporation.

### 2.4. Quantitative Real-Time Polymerase Chain Reaction (qRT-PCR)

The total RNA content was extracted from 100 mg frozen tissues or cells using the TRIzol reagent (12183555, Thermo Fisher Scientific Inc., NY, USA) in a one-step manner. cDNA was synthesized by means of reverse transcription according to the instructions of PrimeScript RT-PCR kits (Takara, Tokyo, Japan). RT-qPCR was performed with SYBR FAST qRT-PCR Master Mix kits (Invitrogen). PCR reaction conditions were as follows: pre-denaturation for 15 min at 95°C, and 40 cycles of denaturation for 30 s at 94°C, annealing for 30 s at 55°C, and extension for 30 s at 72°C. U6 was adopted as the internal control for *miR-613*, and GAPDH was utilized as the internal control for *DNMT3B*, respectively. Following amplification, the dissolution curve was analyzed, and gene expression in each group was calculated using the 2^-*ΔΔ*Ct^ method. Primer sequences are shown in Table 1.

### 2.5. Western Blot Analysis

C666-1 and C666-1R cell homogenates were added with protein lysate (R0278, Sigma-Aldrich, St. Louis, MO, USA) and centrifuged at a rate of 12000 r/min for 20 min at 4°C. Next, the supernatant was subpacked, and total protein concentration in homogenates was detected. Subsequently, 10% sodium dodecyl sulfate separating gel and stacking gel were prepared. Following mixing with the loading buffer, the protein samples were boiled for 5 min at 100°C. Afterwards, the proteins were placed in an ice bath, centrifuged, and equally added to each lane using a micropipette for electrophoresis. Thereafter, the proteins on the gel were transferred onto nitrocellulose membranes, which were blocked with 5% skim milk overnight at 4°C and probed with anti-DNMT3B (ab79822, dilution ratio of 1 : 1000, Abcam Inc., Cambridge, MA, USA), anti-FOXO1 (ab52857, dilution ratio of 1 : 1000, Abcam), anti-STAT1 (ab109461, dilution ratio of 1 : 1000, Abcam), and anti-GAPDH (ab9485, dilution ratio of 1 : 1000, Abcam) at room temperature for 1 h. Later, the membranes were rinsed 3 times with phosphate-buffered saline (PBS) at room temperature, 5 min each. After that, the secondary antibody goat antirabbit IgG H&L (HRP) (ab6721, dilution ratio of 1 : 2000, Abcam) was added for 1-h incubation at room temperature, followed by 3 PBS rinses at room temperature, 5 min each. Afterwards, the membranes were immersed in enhanced chemiluminescence solution (Pierce, Waltham, MA, USA) for 1 min at room temperature. The liquid was absorbed, and the membranes were covered with plastic film and exposed to X-ray film for observation. The gray value ratio of the target band to the internal reference band was regarded as the relative protein expression, with GAPDH serving as the internal reference.

### 2.6. Cell Counting Kit-8 (CCK-8) Assay

Cells (at a density of 5 × 10^3^ cells/mL) were cultured in 96-well plates, with 6 duplicate wells set for each group, and each well was added with 100 *μ*L cell culture medium. After 24 h, cells in the plates well adhered to the wall and then were irradiated with 6-MV X-ray using an accelerator (Elekta Instruments AB, Stockholm, Sweden) at a source-skin distance of 100 cm and a dose rate of 500 cGy/min. The irradiation doses of cells were set to 0, 2, 4, and 8 Gy, respectively. After irradiation, the cells were cultured in an incubator (51020241, Thermo Fisher) for 48 h. Afterwards, 100 *μ*L medium and 10 *μ*L CCK-8 reagent (C0037, Beyotime, Shanghai, China) were successively added to each well under the provided instructions with the CCK-8 kits. After 1–2-h incubation at 37°C, optical density (OD) values at 450 nm were detected using a microplate reader (Multiskan FC, Thermo Fisher). Three parallel wells were set for each group, with the mean value recorded. The cell proliferation curve was plotted with time as the abscissa and OD value as the ordinate.

### 2.7. Colony Formation Assay

Cells were cultured overnight on 6-well plates at a density of 1 × 10^6^ and then treated with 4 Gy irradiation. After 10 days, the cells were fixed with 4% paraformaldehyde, stained with Giemsa stain kit (DM0002, LEAGE, Beijing Leagene Biotechnology, Beijing, China), and then counted under a microscope (CX23, Olympus, Tokyo, Japan).

### 2.8. Flow Cytometry

Cells were rinsed three times with PBS and suspended in the buffer and then subjected to staining with propidium iodide containing 50 *μ*g/mL RNaseA (Sigma-Aldrich) and fluorescein isothiocyanate-AnnexinV (APOAF, Sigma-Aldrich). A FACScan flow cytometer (Beckman Coulter, Fullerton, CA, USA) was utilized for flow cytometric analysis. Data were analyzed using the FlowJo software (Tree Star, San Carlos, CA, USA).

### 2.9. Dual-Luciferase Reporter Gene Assay

The binding sites between *miR-613* and *DNMT3B* were predicted and analyzed with the help of the StarBase database (http://starbase.sysu.edu.cn/index.php). Wild-type or mutant plasmids of *DNMT3B* (wt-*DNMT3B*/mut-*DNMT3B*) were constructed using pmirGLO vectors (GenePharma, Shanghai, China) and subsequently co-transfected with *miR-613* mimic or miR-NC into C666-1 cells in strict accordance with the instructions of the Lipofectamine 2000 reagent (11668, Invitrogen). Luciferase activity was detected 48 h after transfection with the help of a dual-luciferase detection system (Promega, Madison, WI, USA).

### 2.10. Quantitative Real-Time Methylation-Specific PCR (qMSP)

DNA content was extracted from cells under provided instructions of the QIAamp DNA Mini Kit (51304, Qiagen, Hilden, Germany). Bisulfite modification was subsequently carried out in strict accordance with the manufacturer's instructions (Qiagen). DNA sequences were methylated (M) or unmethylated (U) with bisulfite-modified *TIMP3*, and the methylation level of *TIMP3* in cells was detected by qMSP. *TIMP3* methylated or unmethylated primer sequences are illustrated in Table 2 [34].

### 2.11. Statistical Analysis

Data were processed and mapped using the GraphPad Prism 8.0.1 software (GraphPad Software Inc., San Diego, CA, USA). A minimum of three independent cell experiments were carried out per group. Measurement data were presented as mean ± standard deviation (SD). The *t*-test or repeated variance test was utilized for comparisons between two groups, and one-way analysis of variance (ANOVA) was adopted for comparisons between multiple groups, along with Tukey's multiple comparisons test. A value of *p* < 0.05 was regarded as statistically significant.

## 3. Results

### 3.1. *miR-613* Was Poorly Expressed in NPC Tissues and Cells and Associated with NPC Radiosensitivity

**Figure 1:**
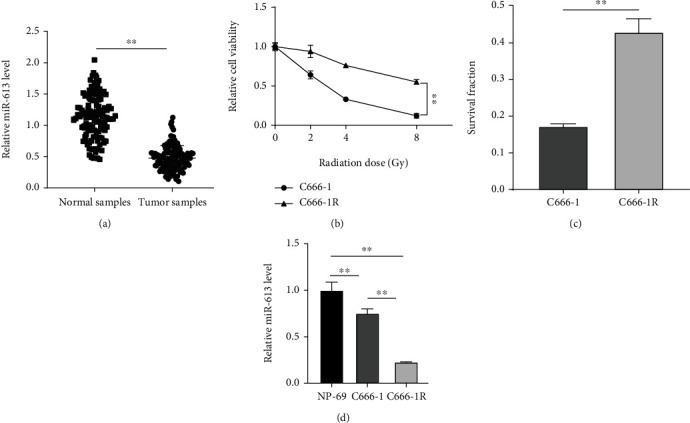
*miR-613* was lowly expressed in NPC tissues and cell lines and was related to radiosensitivity. (a) *miR-613* expression in adjacent normal tissues and tumor tissues of the 121 cases of NPC patients was detected using RT-qPCR; NP-69, NPC cell line C666-1, and the constructed radiation tolerant strain C666-1R were selected for the *in vitro* study. (b) Cell viability of NPC cells after radiation treatment was detected using the CCK-8 method. (c) Cell survival fraction was detected by colony formation assay. (d) *miR-613* expression was detected using RT-qPCR. Three repeated tests were performed. Data were expressed as mean ± SD. *T*-test was used for comparisons between 2 groups in panels (a) and (c), repeated variance test was applied for comparisons between 2 groups in panel (b), and one-way-ANOVA was used for comparisons among multi-groups in panel (d), followed by Tukey's multiple comparisons test. ∗∗*p* < 0.01.

Collection and subsequent RT-qPCR analyses illustrated that the clinical samples of 121 cases of NPC patients presented decreased expression levels of *miR-613* relative to adjacent normal tissues (*p* < 0.01) (Figure 1(a)). To further elucidate the correlation between the expression of *miR-613* and NPC patient clinicopathological characteristics and prognosis, the 121 NPC patients were assigned into the high expression group and low expression groups according to the median value of *miR-613* expression in tumor tissues. There were no significant correlations between the expression of *miR-613* and gender, age, smoking history, and clinical stages, whereas there were significant correlations between miR-613 expression and N classification, T classification, and distant metastasis (all *p* < 0.01) (Table 3), which underscored the involvement of *miR-613* in the progression of NPC.

Accumulating studies have further suggested the usage of *miR-613* as an indicator of the radiosensitivity of cancer cells [49]. To investigate whether *miR-613* could exert a similar role in the radiosensitivity of NPC, NPC cell line C666-1 was selected for *in vitro* experimentation, and the radioresistant NPC cell line C666-1R was obtained by *in vitro* fractionated radiation. CCK-8 was further carried out to detect the cell viability changes in C666-1 and C666-1R cells treated with radiation at the dose of 0, 2, 4, and 8 Gy. With the increase in radiation doses, the cell viability was significantly decreased in both C666-1 < C666-1R cells, and the change was the largest following 4 Gy irradiation (*p* < 0.01) (Figure 1(b)). In addition, C666-1R cells exhibited a higher survival fraction than C666-1 cells under 4 Gy radiation (*p* < 0.01) (Figure 1(c)). Furthermore, *miR-613* level patterns in cells were detected using RT-qPCR, which revealed that compared with NP-69 cells, *miR-613* was down-regulated in C666-1 and C666-1R cells; and the expression in tolerant C666-1R cells was relatively lower than that of the C666-1 cells (*p* < 0.01) (Figure 1(d)). Together, these findings highlighted that *miR-613* might be involved in the regulation of NPC cell radiosensitivity.

### 3.2. Overexpression of *miR-613* Improved NPC Cell Radiosensitivity

**Figure 2:**
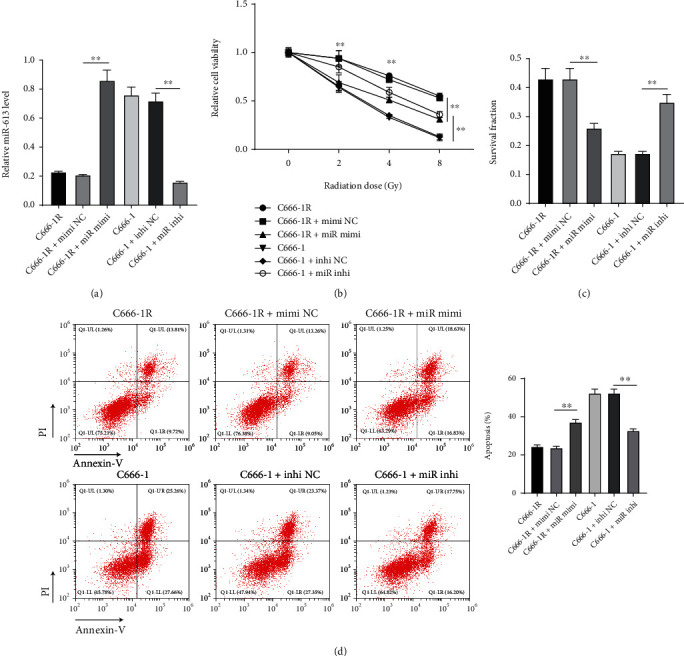
Overexpression of *miR-613* increased NPC cell radiosensitivity. (a) *miR-613* expression in cells was detected using RT-qPCR. (b) Cell viability was detected using the CCK-8 method. (c) Cell survival fraction was detected by colony formation assay. (d) Cell apoptosis was detected by flow cytometry. Three repeated tests were performed. Data were expressed as mean ± SD and analyzed using one-way ANOVA and Tukey's multiple comparisons test. ∗*p* < 0.05, ∗∗*p* < 0.01.

To further elaborate on the role of *miR-613* in NPC cell radiosensitivity, we transfected C666-1R cells with *miR-613* mimics to overexpress *miR-613* expression and transfected C666-1 cells with *miR-613* inhibitor to down-regulate *miR-613* expression (*p* < 0.01) (Figure 2(a)). Subsequent experimentation demonstrated that C666-1R cell viability was decreased under 0, 2, 4, and 8 Gy radiation after *miR-613* overexpression, the surviving fraction was decreased, and the apoptosis rate was increased under 4 Gy radiation (all *p* < 0.01); meanwhile, C666-1 cell viability was increased under 0, 2, 4, and 8 Gy radiation after *miR-613* down-regulation, and the surviving fraction was increased, and the apoptosis rate was decreased under 4 Gy radiation (Figures 2(b)–2(d)). Overall, these findings indicated that overexpression of *miR-613* exerted an enhancing effect on NPC cell radiosensitivity.

*miR-613* increased NPC radiosensitivity by targeting *DNMT3B.*

**Figure 3:**
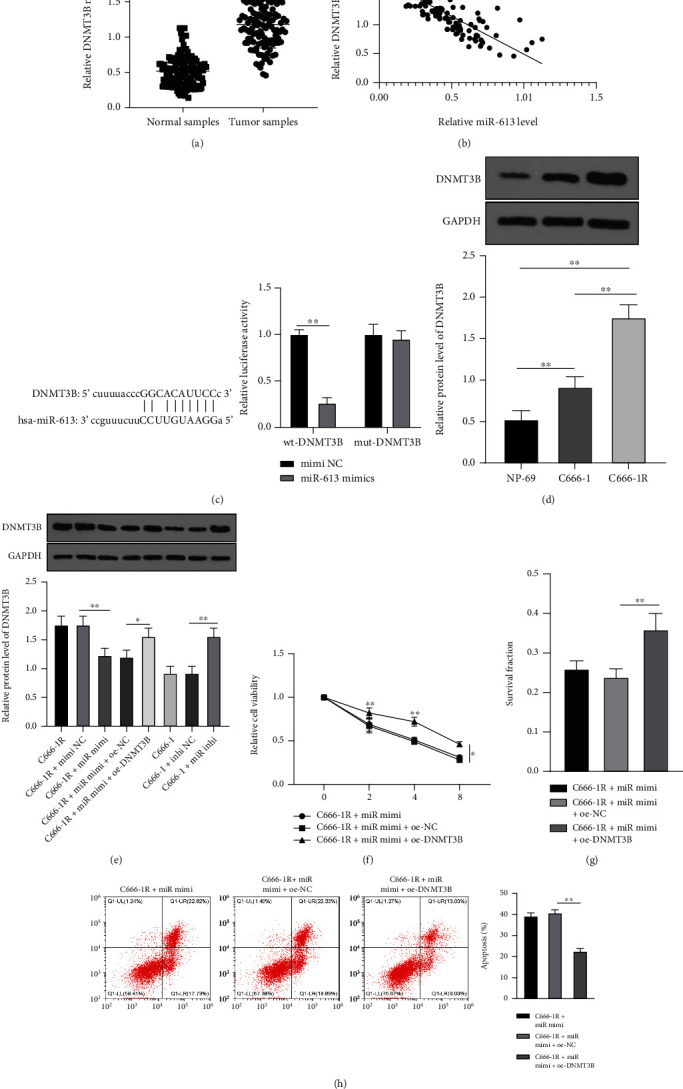
*miR-613* increased NPC cell radiosensitivity by inhibiting *DNMT3B*. (a) mRNA levels of *DNMT3B* in cancer tissues and adjacent tissues of 121 NPC patients were detected by RT-qPCR. (b) Correlation between *DNMT3B* and *miR-613*. (c) Binding relationship between *DNMT3B* and *miR-613* was verified by dual-luciferase assay. (d–e) Protein level of *DNMT3B* in cells was detected by Western blot. (f) Cell viability of C666-1R cells was detected using the CCK-8 method. (g) Cell clone formation and apoptosis in C666-1R cells were detected by colony formation and (h) flow cytometry. Three repeated tests were performed. Data were expressed as mean ± SD, and data in panels (a) and (c) were analyzed using independent *t* test; data in panels (d), (e), (g), and (h) were analyzed using one-way ANOVA, followed by Tukey's test; and data in panel (f) were analyzed using repeated variance test. ∗*p* < 0.05, ∗∗*p* < 0.01.

*DNMT3B* was associated with NPC radiosensitivity [37]. Further detection illustrated that *DNMT3B* was highly expressed in NPC tissues (Figure 3(a), *p* < 0.01), and negatively correlated with *miR-613* expression (Figure 3(b)). Subsequently, the targeted binding sites between *miR-613* and *DNMT3B* were predicted using the StarBase database and verified with a dual-luciferase assay (Figure 3(c)). Moreover, the results of Western blot elicited higher protein levels of *DNMT3B* in C666-1R cell line relative to those in NP-69 and C666-1 cells (*p* < 0.01) (Figure 3(d)). After *miR-613* overexpression, the protein levels of *DNMT3B* in C666-1R cells were diminished; whereas after *miR-613* knockdown, the protein levels of *DNMT3B* in C666-1 cells were enhanced (Figure 3(e), *p* < 0.01). These findings suggested that *miR-613* was capable of targeting *DNMT3B*. Subsequently, C666-1R cells were co-transfected with *miR-613* mimics and oe-*DNMT3B*, followed by analyses of cell radiosensitivity. Subsequent results demonstrated augmented protein levels of *DNMT3B* in C666-1R cells (Figure 3(e), *p* < 0.05). Under 0, 2, 4, and 8 Gy radiation, cell viability was increased, cell surviving fraction under 4 Gy radiation was increased, and apoptosis was reduced (all *p* < 0.05) (Figures 3(f)–3(h)). Altogether, these findings indicated that *miR-613* enhanced NPC radiosensitivity *via* inhibition of *DNMT3B*.

### 3.3. *miR-613* Reduced *TIMP3* Methylation by Inhibiting *DNMT3B*

**Figure 4:**
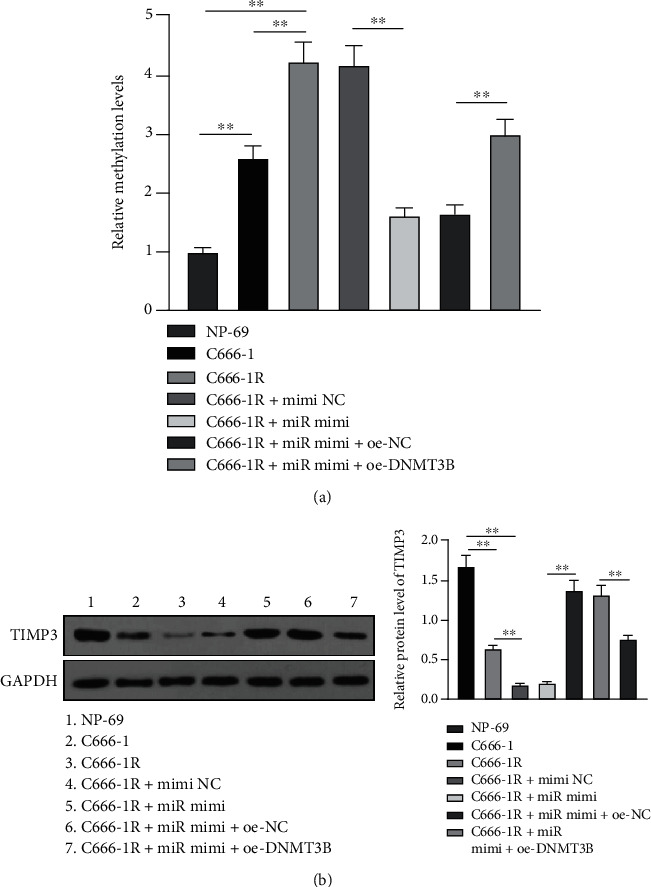
*miR-613* reduced *TIMP3* methylation by inhibiting *DNMT3B*. (a) The methylation level of *TIMP3* in cells was detected by qMSP. (b) The protein level of *TIMP3* was detected by Western blot. Cell experiment was repeated 3 times, and the data were expressed as mean ± SD and analyzed using one-way ANOVA, followed by Tukey's multiple comparisons test. ∗∗*p* < 0.01.

*TIMP3* was previously established to be lowly expressed in NPC tissues [42], and *TIMP3* methylation is a common occurrence in many cancers [34, 43, 44]. *DNMT3B* further represents a major DNA methyltransferase regulated by *miR-613*. To explore whether *miR-613* reduced the methylation level of *TIMP3* in NPC cells by inhibiting *DNMT3B* expression, *TIMP3* methylation level and its protein level patterns were detected in cells by qMSP and Western blot. Subsequent results illustrated that compared with NP-69 cells, *TIMP3* methylation levels were elevated, and *TIMP3* protein levels were suppressed in C666-1 and C666-1R cells; meanwhile, compared with C666-1 cells, *TIMP3* methylation levels were enhanced, and *TIMP3* protein levels were diminished in C666-1R cells, whereas after overexpression of *miR-613*, *TIMP3* methylation levels were decreased, and *TIMP3* protein levels were elevated in C666-1R cells. After further overexpression of *miR-613* and overexpression of *DNMT3B*, *TIMP3* methylation levels were enhanced, and *TIMP3* protein levels were repressed in C666-1R cells (Figures 4(a) and 4(b), *p* < 0.01). Altogether, these findings highlighted that *miR-613* reduced *TIMP3* methylation levels by inhibiting the expression of *DNMT3B*.

### 3.4. *miR-613* Enhanced Radiosensitivity by Inhibiting the *DNMT3B*/*TIMP3*/STAT1/FOXO1 Pathway

**Figure 5:**
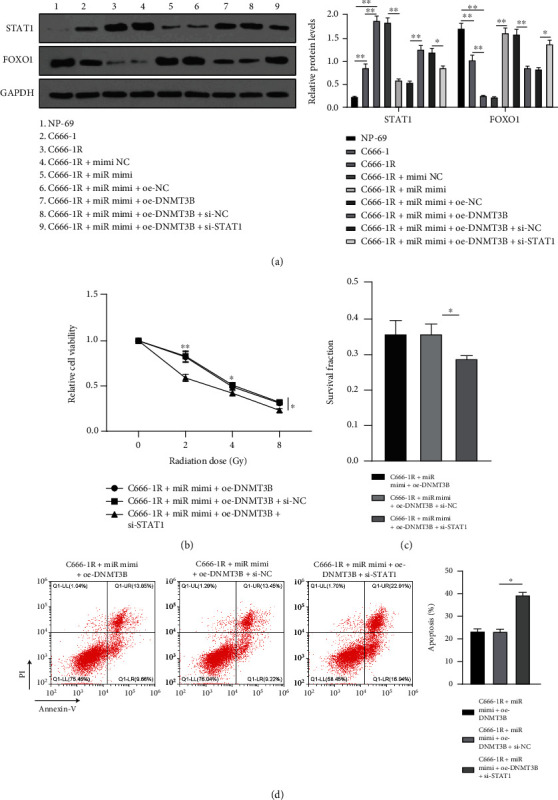
*miR-613* enhanced NPC radiosensitivity by inhibiting the *DNMT3B*/*TIMP3*/STAT1/FOXO1 pathway. (a) Protein levels of STAT1 and FOXO1 in cells were detected by Western blot. (b) Cell viability changes of cells were detected using the CCK-8 method. (c) Cell survival fraction of cells was detected by cell colony formation assay. (d) Cell apoptosis was detected by flow cytometry. Three independent repeated tests were performed. Data were expressed as mean ± SD and analyzed using one-way ANOVA and Tukey's multiple comparisons test. ∗*p* < 0.05, ∗∗*p* < 0.01.

*DNMT3B* is known to influence breast cancer development *via* regulation of the STAT1/FOXO1 pathway [34]. Moreover, STAT1 exerts a regulatory effect on the radiosensitivity of a variety of cancer cells [50–52]. To further explore whether *miR-613* participated in the regulation of NPC radiosensitivity by inhibiting *DNMT3B*/*TIMP3* and diminishing the activation of the STAT1/FOXO1 pathway, the protein level patterns of STAT1 and FOXO1 in NP-69, C666-1, and C666-1R were detected by Western blot. Subsequent results demonstrated that compared with NP-69 cells, the protein levels of STAT1 in C666-1 and C666-1R cells were increased, with C666-1R presenting with higher STAT1 levels than those in C666-1, and the protein levels of FOXO1 were decreased, with C666-1R presenting with lower STAT1 levels than those in C666-1 (Figure 5(a), *p* < 0.01). Moreover, C666-1R cells were co-transfected with *miR-613* mimic, oe-*DNMT3B*, and si-STAT1. It was found that STAT1 levels were noticeably reduced, and FOXO1 levels were markedly elevated in C666-1R cells after *miR-613* overexpression, while these trends were reversed in CNE-2R cells after *miR-613* overexpression and *DNMT3B* overexpression. Lastly, STAT1 protein levels were significantly decreased, and FOXO1 protein levels were enhanced in C666-1R cells after transfection with *miR-613* mimics, oe-*DNMT3B*, and si-STAT1, whereas C666-1R presented with decreased cell viability, reduced cell surviving fraction under 4 Gy radiation, and increased cell apoptosis under 2, 4, and 8 Gy radiation (Figures 5(a)–5(d), *p* < 0.05). Altogether, these findings highlighted that *miR-613* increased the radiosensitivity of NPC cells by inhibiting the *DNMT3B*/*TIMP3*/STAT1/FOXO1 pathway.

## 4. Discussion

NPC represents a common head and neck malignancy arising from the epithelium of the nasopharynx, associated with low survival rates in certain endemic areas [1]. Meanwhile, the last decade has witnessed the emergence of miRNAs as key regulators in diverse biological functions and disease progression [53]. Herein, the current study set out to elucidate the potential roles of *miR-613* in NPC cell radiosensitivity, and our findings highlighted that *miR-613* inhibited *DNMT3B*, reduced *TIMP3* methylation, and increased *TIMP3* protein level, thereby inhibiting the downstream STAT1/FOXO1 pathway and augmenting the radiosensitivity of NPC cells.

Our study is not the first of its kind to suggest the involvement of miRNA regulation of radiosensitivity in NPC cells [54]. Initial findings in our study demonstrated that *miR-613* was poorly expressed in NPC tissues, and the aberrant expression of *miR-613* was associated with N classification, T classification, and distant metastasis. Additional experimentation with increasing radiation doses revealed that there was a significant decrease in the cell viability of parental NPC cell line C666-1 and the radioresistant cell line C666-1R, and the change was the largest under 4 Gy irradiation, and C666-1 < C666-1R; meanwhile, C666-1R exhibited a higher survival fraction than C666-1 under 4 Gy radiation. Compared with NP-69 cells, *miR-613* expression levels were down-regulated in C666-1 and C666-1R cells, while relative to the C666-1 cells, *miR-613* expression levels were also diminished in the radiotolerant C666-1R cells. In accordance with our data, previous studies have documented that *miR-613* expression is lowered in NPC cell lines and tissues [29]. Similarly, *miR-613* was previously reported to exert effects on the cell radiosensitivity of cancers, such as nonsmall cell lung cancer, while also being implicated in cisplatin resistance of glioma [49, 55]. Altogether, these findings and evidence conclusively highlight the involvement of *miR-613* in NPC cell radiosensitivity regulation.

Accumulating reports have further indicated the use of *miR-613* as an indicator of the radiosensitivity of cancer cells [49]. To further elaborate on the role of *miR-613* in NPC cell radiosensitivity, we overexpressed *miR-613* expression in C666-1R cells and knocked down *miR-613* expression in C666-1 cells. Subsequent experimentation demonstrated that following overexpression of *miR-613*, C666-1R cell viability was reduced under 0, 2, 4, and 8 Gy radiation, and the surviving fraction was repressed, and apoptosis rate was elevated under 4 Gy radiation, whereas C666-1 cells with silenced *miR-613* presented with the opposite trends. Consistently, a prior report suggested that hepatoma cells with overexpressed *miR-613* were more sensitive to sorafenib or cisplatin treatment [56]. Together, our findings in conjunction with existing evidence highlight that overexpression of *miR-613* augments NPC cell radiosensitivity.

Another key component of our study, the *DNMT3B* gene is known to play roles in tumorigenesis, metastasis, progression, and NPC cell radiosensitivity [33, 35–37]. Further detection revealed that *DNMT3B* was highly expressed in NPC tissues and negatively correlated with *miR-613*. We further validated the targeting relationship between *miR-613* and *DNMT3B* using a dual-luciferase assay and came across higher *DNMT3B* protein levels in C666-1R cell line relative to NP-69 and C666-1 cells. In addition, overexpression of *miR-613* led to diminished *DNMT3B* protein levels in C666-1R cells, while knockdown of *miR-613* brought about an increase in *DNMT3B* protein levels in C666-1 cells, which underscores that *miR-613* targeted *DNMT3B*. In accordance with our data, elevated *DNMT3B* expression in NPC tissues has been reported before [37]. Furthermore, we verified the role of the *miR-613*/*DNMT3B* axis in NPC cell radiosensitivity that after co-transfection with *miR-613* mimics and oe-*DNMT3B*, there was an increase in *DNMT3B* protein levels in C666-1R cells. Meanwhile, under 0, 2, 4, and 8 Gy radiation, cell viability was augmented, survival fraction was stimulated, and apoptosis was reduced under 4 Gy radiation. Consistently, *DNMT3B* induced by radiation was previously indicated to promote NPC radioresistance through methylation of p21 and p53 [37]. Likewise, another study indicated that overexpression of DNMT3B contributes to radiotherapy failure, such that its inhibition represents a promising radiosensitizing strategy for patients with metastatic or recurrent rhabdomyosarcoma tumors [57]. Exosomal *miR-613* also exerts an inhibitory effect on lung cancer growth, while promoting the sensitivity to cisplatin [58]. Silencing has-circ0000199 enhanced breast cancer chemosensitivity by promoting *miR-613* expression [59]. Overall, these findings and data indicate that *miR-613* enhances NPC radiosensitivity by targeting *DNMT3B*.

Furthermore, various novel studies have come across *TIMP3* methylation in a myriad of cancers [34, 43, 44]. Herein, our results illustrated that *TIMP3* methylation levels were augmented and *TIMP3* protein levels were suppressed in C666-1 and C666-1R cells, with C666-1R cells showing the more obvious alteration; meanwhile, after *miR-613* overexpression, *TIMP3* methylation levels were suppressed, and *TIMP3* protein levels were facilitated in C666-1R cells, whereas further *DNMT3B* overexpression reversed the above trends in C666-1R cells. Consistent lowly expressed *TIMP3* was previously reported in NPC tissues [42], while knockdown of *TIMP3* methylation was associated with alleviation of breast cancer [34]. In short, these findings and data make it plausible to suggest that *miR-613* reduces *TIMP3* methylation levels by inhibiting *DNMT3B*.

The last focus of our study, STAT1, is widely established to regulate cell radiosensitivity in various cancers [50–52]. To investigate whether miR-613 regulated NPC radiotherapy by inhibiting *DNMT3B*/*TIMP3* and the STAT1/FOXO1 pathway, we detected STAT1 and FOXO1 protein levels in NP-69, C666-1, and C666-1R cells, and discovered that STAT1 protein levels were promoted and FOXO1 protein levels were decreased in C666-1 and C666-1R cells, with C666-1R cells showing the more obvious alteration. Moreover, STAT1 levels were markedly diminished, and FOXO1 levels were augmented in C666-1R cells after *miR-613* overexpression, which were reversed after further *DNMT3B* overexpression, and restored by further silencing STAT1, in addition to decreased cell viability and surviving fraction and enhanced cell apoptosis in C666-1R cells. Consistently, STAT1 silencing has previously been shown to sensitize NPC cell line CNE-2R radioresistant to radiotherapy [60]. Moreover, human glioma cell radiosensitivity can similarly be enhanced by LITAF *via* the FOXO1 pathway [61]. Unsurprisingly, there is prior evidence to suggest that *DNMT3B* affects the progression of breast cancer by targeting the STAT1/FOXO1 pathway [62]. Altogether, these findings illustrated that *miR-613* diminishes the radiosensitivity of NPC cells by inhibiting the *DNMT3B*/*TIMP3 via* activation of the STAT1/FOXO1 pathway for the first time.

In summary, the current study highlighted that *miR-613* inhibited *DNMT3B*, reduced *TIMP3* methylation, and increased *TIMP3* protein level, thus inhibiting the downstream STAT1/FOXO1 pathway and enhancing the radiosensitivity of NPC cells. However, we only carried out the relevant mechanism research in NPC cell line, and our findings warrant *in vivo* validation experiments in future studies.

## Figures and Tables

**Table 1:** PCR primer sequences.

Gene	Primer sequences (5′-3′)
*miR-613*	F 5′-AGGAATGTTCCTTCTTTGCC-3′
	R 5′-CAGTGCGTGTCGTGGAGT-3′
*DNMT3B*	F 5′-GTCAGGCGCTGACATAGTGA-3′
	R 5′-TCATGACTGGCAGAGGACAG-3′
*GAPDH*	F 5′-GGTGAAGGTCGGAGTCAACGG-3′
	R 5′-CCTGGAAGATGGTGATGGGATT-3′
*U6*	F 5′-CTCGCTTCGGCAGCACA-3′
	R 5′-AACGCTTCACGAATTTGCGT-3′

**Table 2:** TIMP3 methylated (M) or unmethylated (U) primer sequences.

Primer type	Primer sequences
M	Forward: 5′-TCGAGAGATAGAAATATTTTTACGA-3′
	Reverse: 5′-TAACATTAAAACAACAAAAACCGAA-3′
U	Forward: 5′-TTGAGAGATAGAAATATTTTTATGA-3′
	Reverse: 5′-TAACATTAAAACAACAAAAACCAAA-3′

**Table 3:** Correlation between NPC patient clinical features and *miR-613* expression.

Characteristics		Expression of *miR-613* (%)		*p*
		High expression	Low expression	
		(*N* = 60)	(*N* = 61)	
Gender	Male	35 (46.1%)	41 (53.9%)	0.3504
	Female	25 (55.6%)	20 (44.4%)	
Age (y)	≥ 50	36 (58.1%)	26 (41.9%)	0.0696
	< 50	24 (40.7%)	35 (59.3%)	
Smoking	Yes	25 (61.0%)	16 (39.0%)	0.0858
	No	35 (43.8%)	45 (56.2%)	
T classification	T1-T2	36 (39.1%)	56 (60.9%)	< 0.0001
	T3-T4	24 (82.8%)	5 (17.2%)	
N classification	N0-N1	32 (62.7%)	19 (37.3%)	0.0169
	N2-N3	28 (40.0%)	42 (60.0%)	
Distant metastasis	Yes	12 (29.3%)	29 (70.7%)	0.002
	No	48 (60.0%)	32 (40.0%)	
Clinical stage	I-II	26 (63.4%)	20 (36.6%)	0.2643
	III-IV	34 (48.8%)	41 (51.2%)	

## Data Availability

The data used to support the findings of this study are available from the corresponding author upon request.

## References

[1] Paul P., Deka H., Malakar A. K., Halder B., Chakraborty S. (2018). Nasopharyngeal carcinoma: understanding its molecular biology at a fine scale. *European Journal of Cancer Prevention*.

[2] Zhang L., MacIsaac K. D., Zhou T. (2017). Genomic analysis of nasopharyngeal carcinoma reveals TME-based subtypes. *Molecular Cancer Research*.

[3] Chen Y. P., Chan A. T. C., Le Q. T., Blanchard P., Sun Y., Ma J. (2019). Nasopharyngeal carcinoma. *The Lancet*.

[4] Petersson F. (2015). Nasopharyngeal carcinoma: a review. *Seminars in Diagnostic Pathology*.

[5] Roy Chattopadhyay N., Das P., Chatterjee K., Choudhuri T. (2017). Higher incidence of nasopharyngeal carcinoma in some regions in the world confers for interplay between genetic factors and external stimuli. *Drug discoveries & therapeutics*.

[6] Sun X. S., Li X. Y., Chen Q. Y., Tang L. Q., Mai H. Q. (2019). Future of radiotherapy in nasopharyngeal carcinoma. *The British Journal of Radiology*.

[7] Tan Z., Xiao L., Tang M. (2018). Targeting CPT1A-mediated fatty acid oxidation sensitizes nasopharyngeal carcinoma to radiation therapy. *Theranostics*.

[8] Gui S. J., Ding R. L., Wan Y. P. (2020). Knockdown of annexin VII enhances nasopharyngeal carcinoma cell radiosensitivity in vivo and in vitro. *Cancer Biomarkers*.

[9] Saliminejad K., Khorram Khorshid H. R., Soleymani Fard S., Ghaffari S. H. (2019). An overview of microRNAs: biology, functions, therapeutics, and analysis methods. *Journal of Cellular Physiology*.

[10] Kang M., Xiao J., Wang J. (2016). MiR-24 enhances radiosensitivity in nasopharyngeal carcinoma by targeting SP1. *Cancer Medicine*.

[11] Lin T., Zhou F., Zhou H., Pan X., Sun Z., Peng G. (2015). MicroRNA-378g enhanced radiosensitivity of NPC cells partially by targeting protein tyrosine phosphatase SHP-1. *International Journal of Radiation Biology*.

[12] Zhao L., Tang M., Hu Z. (2015). miR-504 mediated down-regulation of nuclear respiratory factor 1 leads to radio-resistance in nasopharyngeal carcinoma. *Oncotarget*.

[13] Ambros V. (2004). The functions of animal microRNAs. *Nature*.

[14] Min A., Zhu C., Peng S., Rajthala S., Costea D. E., Sapkota D. (2015). MicroRNAs as important players and biomarkers in oral carcinogenesis. *BioMed Research International*.

[15] Crimi S., Falzone L., Gattuso G. (2020). Droplet digital PCR analysis of liquid biopsy samples unveils the diagnostic role of hsa-miR-133a-3p and hsa-miR-375-3p in oral cancer. *Biology*.

[16] Peruhova M., Peshevska-Sekulovska M., Krastev B. (2020). What could microRNA expression tell us more about colorectal serrated pathway carcinogenesis?. *World Journal of Gastroenterology*.

[17] Spadaccino F., Gigante M., Netti G. S. (2021). The ambivalent role of miRNAs in carcinogenesis: involvement in renal cell carcinoma and their clinical applications. *Pharmaceuticals*.

[18] Di Leva G., Garofalo M., Croce C. M. (2014). MicroRNAs in cancer. *Annual Review of Pathology*.

[19] Xie F., Xiao W., Tian Y., Lan Y., Zhang C., Bai L. (2021). MicroRNA-195-3p inhibits cyclin dependent kinase 1 to induce radiosensitivity in nasopharyngeal carcinoma. *Bioengineered*.

[20] Yu H., Duan P., Zhu H., Rao D. (2017). miR-613 inhibits bladder cancer proliferation and migration through targeting SphK1. *American Journal of Translational Research*.

[21] Ding D., Hou R., Gao Y., Feng Y. (2018). miR-613 inhibits gastric cancer progression through repressing brain derived neurotrophic factor. *Experimental and Therapeutic Medicine*.

[22] Jiang X., Wu J., Zhang Y. (2018). MiR-613 functions as tumor suppressor in hepatocellular carcinoma by targeting YWHAZ. *Gene*.

[23] Zhang Y., Zhu X., Zhu X. (2017). MiR-613 suppresses retinoblastoma cell proliferation, invasion, and tumor formation by targeting E2F5. *Tumour Biology*.

[24] Liu H., Chen K., Wang L. (2019). miR-613 inhibits Warburg effect in gastric cancer by targeting PFKFB2. *Biochemical and Biophysical Research Communications*.

[25] Sang Q., Liu X., Sun D. (2018). Role of miR-613 as a tumor suppressor in glioma cells by targeting SOX9. *Oncotargets and Therapy*.

[26] Huang Y., Zhang H., Wang L. (2021). MiR-613 inhibits the proliferation, migration, and invasion of papillary thyroid carcinoma cells by directly targeting TAGLN2. *Cancer Cell International*.

[27] Cai H., Yao J., An Y. (2017). LncRNA HOTAIR acts a competing endogenous RNA to control the expression of notch3 via sponging miR-613 in pancreatic cancer. *Oncotarget*.

[28] Chen W., Du M., Hu X. (2020). Long noncoding RNA cytoskeleton regulator RNA promotes cell invasion and metastasis by titrating miR-613 to regulate ANXA2 in nasopharyngeal carcinoma. *Cancer Medicine*.

[29] Dai W., Shi Y., Hu W., Xu C. (2022). Long noncoding RNA FAM225B facilitates proliferation and metastasis of nasopharyngeal carcinoma cells by regulating miR-613/CCND2 axis. *Bosnian Journal of Basic Medical Sciences*.

[30] Gao R., Feng Q., Tan G. (2019). microRNA-613 exerts anti-angiogenic effect on nasopharyngeal carcinoma cells through inactivating the AKT signaling pathway by down-regulating FN1. *Bioscience Reports*.

[31] Lakshminarasimhan R., Liang G. (2016). The role of DNA methylation in cancer. *Advances in Experimental Medicine and Biology*.

[32] Giambò F., Leone G. M., Gattuso G. (2021). Genetic and epigenetic alterations induced by pesticide exposure: integrated analysis of gene expression, microRNA expression, and DNA methylation datasets. *International journal of environmental research and public health*.

[33] Chen L. H., Hsu W. L., Tseng Y. J., Liu D. W., Weng C. F. (2016). Involvement of DNMT 3B promotes epithelial-mesenchymal transition and gene expression profile of invasive head and neck squamous cell carcinomas cell lines. *BMC Cancer*.

[34] Li W., Yi J., Zheng X. (2018). miR-29c plays a suppressive role in breast cancer by targeting the TIMP3/STAT1/FOXO1 pathway. *Epigenetics*.

[35] Linhart H. G., Lin H., Yamada Y. (2007). Dnmt3b promotes tumorigenesis in vivo by gene-specific de novo methylation and transcriptional silencing. *Genes & Development*.

[36] Yang Y. C., Tang Y. A., Shieh J. M., Lin R. K., Hsu H. S., Wang Y. C. (2014). DNMT3B overexpression by deregulation of FOXO3a-mediated transcription repression and MDM2 overexpression in lung cancer. *Journal of Thoracic Oncology*.

[37] Wu C., Guo E., Ming J. (2020). Radiation-induced DNMT3B promotes radioresistance in nasopharyngeal carcinoma through methylation of p53 and p21. *Molecular Therapy-Oncolytics*.

[38] Huang H. L., Liu Y. M., Sung T. Y. (2019). TIMP3 expression associates with prognosis in colorectal cancer and its novel arylsulfonamide inducer, MPT0B390, inhibits tumor growth, metastasis and angiogenesis. *Theranostics*.

[39] Zhang Z., Wang J., Wang X., Song W., Shi Y., Zhang L. (2018). MicroRNA-21 promotes proliferation, migration, and invasion of cervical cancer through targeting TIMP3. *Archives of Gynecology and Obstetrics*.

[40] Qin S., Zhu Y., Ai F. (2014). MicroRNA-191 correlates with poor prognosis of colorectal carcinoma and plays multiple roles by targeting tissue inhibitor of metalloprotease 3. *Neoplasma*.

[41] Chen J., Gu Y., Shen W. (2017). MicroRNA-21 functions as an oncogene and promotes cell proliferation and invasion via TIMP3 in renal cancer. *European Review for Medical and Pharmacological Sciences*.

[42] Zhao Y., Gu X., Wang Y. (2020). MicroRNA-103 promotes nasopharyngeal carcinoma through targeting TIMP-3 and the Wnt/*β*‐catenin pathway. *The Laryngoscope*.

[43] Lim Y., Wan Y., Vagenas D. (2016). Salivary DNA methylation panel to diagnose HPV-positive and HPV-negative head and neck cancers. *BMC Cancer*.

[44] Guilleret I., Losi L., Chelbi S. T. (2016). DNA methylation profiling of esophageal adenocarcinoma using methylation ligation-dependent macroarray (MLM). *Biochemical and Biophysical Research Communications*.

[45] Zhang Z., Huo H., Liao K. (2018). RPA1 downregulation enhances nasopharyngeal cancer radiosensitivity _via_ blocking RAD51 to the DNA damage site. *Experimental Cell Research*.

[46] Shepherd F. A., Crowley J., Van Houtte P., Postmus P. E., Carney D., Chansky K. (2007). The International Association for the Study of Lung Cancer lung cancer staging project: proposals regarding the clinical staging of small cell lung cancer in the forthcoming (seventh) edition of the tumor, node, metastasis classification for lung cancer. *Journal of Thoracic Oncology*.

[47] Wan F. Z., Chen K. H., Sun Y. C. (2020). Exosomes overexpressing miR-34c inhibit malignant behavior and reverse the radioresistance of nasopharyngeal carcinoma. *Journal of Translational Medicine*.

[48] Du T., Jiang J., Chen Y. (2021). MiR-138-1-3p alters the stemness and radiosensitivity of tumor cells by targeting CRIPTO and the JAK2/STAT3 pathway in nasopharyngeal carcinoma. *Annals of Translational Medicine*.

[49] Chen X., Xu Y., Liao X. (2016). Plasma miRNAs in predicting radiosensitivity in non-small cell lung cancer. *Tumour Biology*.

[50] Hui Z., Tretiakova M., Zhang Z. (2009). Radiosensitization by inhibiting STAT1 in renal cell carcinoma. *International Journal of Radiation Oncology • Biology • Physics*.

[51] Khodarev N. N., Beckett M., Labay E., Darga T., Roizman B., Weichselbaum R. R. (2004). STAT1 is overexpressed in tumors selected for radioresistance and confers protection from radiation in transduced sensitive cells. *Proceedings of the National Academy of Sciences of the United States of America*.

[52] Zhu H., Wang Z., Xu Q. (2012). Inhibition of STAT1 sensitizes renal cell carcinoma cells to radiotherapy and chemotherapy. *Cancer Biology & Therapy*.

[53] Tan L. P., Tan G. W., Sivanesan V. M. (2020). Systematic comparison of plasma EBV DNA, anti-EBV antibodies and miRNA levels for early detection and prognosis of nasopharyngeal carcinoma. *International Journal of Cancer*.

[54] Chang L. H., Yao Z. Z., Bao H. W. (2021). miR-18a enhances the radiosensitivity of nasopharyngeal carcinoma cells through inducing autophagy. *Zhonghua Er Bi Yan Hou Tou Jing Wai Ke Za Zhi*.

[55] Ma Y., Zhou G., Li M. (2018). Long noncoding RNA DANCR mediates cisplatin resistance in glioma cells via activating AXL/PI3K/Akt/NF-*κ*B signaling pathway. *Neurochemistry International*.

[56] Li B., Liu D., Yang P., Li H. Y., Wang D. (2019). miR-613 inhibits liver cancer stem cell expansion by regulating SOX9 pathway. *Gene*.

[57] Camero S., Vitali G., Pontecorvi P. (2021). DNMT3A and DNMT3B targeting as an effective radiosensitizing strategy in embryonal rhabdomyosarcoma. *Cells*.

[58] Zhai J. X., Zhang Z. X., Feng Y. J. (2012). PDTC attenuate LPS-induced kidney injury in systemic lupus erythematosus-prone MRL/lpr mice. *Molecular Biology Reports*.

[59] Li H., Xu W., Xia Z. (2021). Hsa_circ_0000199 facilitates chemo-tolerance of triple-negative breast cancer by interfering with miR-206/613-led PI3K/Akt/mTOR signaling. *Aging*.

[60] Qu S., Guo Y., Huang S. T., Zhu X. D. (2018). Inhibition of STAT1 sensitizes radioresistant nasopharyngeal carcinoma cell line CNE-2R to radiotherapy. *Oncotarget*.

[61] Huang C., Chen D., Zhu H., Lv S., Li Q., Li G. (2019). LITAF enhances radiosensitivity of human glioma cells via the FoxO1 pathway. *Cellular and Molecular Neurobiology*.

[62] Zhang P., Xin X., Fang L. (2017). HMGB1 mediates Aspergillus fumigatus-induced inflammatory response in alveolar macrophages of COPD mice via activating MyD88/NF-kappaB and syk/PI3K signalings. *International Immunopharmacology*.

